# NADC30-like Strain of Porcine Reproductive and Respiratory Syndrome Virus, China

**DOI:** 10.3201/eid2112.150360

**Published:** 2015-12

**Authors:** Lei Zhou, Zichun Wang, Yuping Ding, Xinna Ge, Xin Guo, Hanchun Yang

**Affiliations:** Author affiliation: China Agricultural University, Beijing, China

**Keywords:** porcine reproductive and respiratory syndrome, porcine reproductive and respiratory syndrome virus, PRRSV, viruses, NADC30-like strain, pigs, outbreaks, zoonoses, China

**To the Editor:** Porcine reproductive and respiratory syndrome (PRRS), which is characterized by reproductive failure in sows and respiratory disease in pigs of all ages, is a viral disease with serious economic consequences for the global pork industry (*1*). PRRS virus (PRRSV), the causative agent of this disease, was identified in Europe in 1991 and the United States in 1992 (*2**,**3*). PRRSV is an enveloped, positive-strand RNA virus of the family *Arteriviridae*. This virus is divided into European genotype 1 and North American genotype 2. Emerging novel PRRSV strains have caused many outbreaks of severe PRRS (*4**–**7*). We report emergence of a novel PRRSV (NADC30-like) in China that is genetically similar to the NADC30 strain isolated in the United States in 2008 (*8*).

During August–December 2014, severe outbreaks of PRRS were observed on 7 intensive pig farms in Beijing, Tianjing, Shanxi, Henan, and Zhejing, China. Pregnant sows had abortions and stillbirth and piglets had respiratory disorders (case-fatality rate 30%–50%).

A total of 58 tissue samples from stillborn piglets, serum samples from diseased sows and piglets, and lungs and lymph nodes of dead piglets were tested for viral RNA by using reverse transcription PCR and primers specific for PRRSV open reading frame (ORF) 7, which encodes nucleocapsid protein, as described (*9*). Viral RNA was detected in 63.8% (4/7, 7/13, 5/5, 4/4, 5/10, 6/6, and 6/13 for the 7 farms, respectively) of samples tested.

All virus-positive lung samples were then used to amplify the entire ORF5 gene, which encodes major envelope glycoprotein 5 and is one of the most variable regions in the PRRSV genome. Amplified fragments were sequenced to analyze variation of PRRSV as described (*10*). 

Comparative analyses of sequences showed that amplified ORF5s of viruses isolated on an individual farm had 100% identities and amplified ORF5s of viruses from 7 farms had 89.7%–97.7% nucleotide identities (88.6%–98.0% for deduced amino acids) with each other (GenBank accession nos. KP861625–31) and higher nucleotide (92.2%–97.0%) and amino acid (91.5%–96.5%) identities with NADC30. The ≈10% amino acid divergence among ORF5s from the 7 farms suggests possible variation of NADC30 during its transmission. These viruses had lower nucleotide (84.9%–87.6%) and amino acid (84.1%–88.6%) identities with representative PRRSV strains from China, including CH1a, HB-1(sh)/2002, HB-2(sh)/2002, and JXwn06, and lower nucleotide (85.1%–86.7%) and amino acid (82.1%–86.1%) identities with VR-2332.

A strain of PRRSV (CHsx1401) was isolated from a lung sample by using porcine pulmonary alveolar macrophages. Third-passage viral cultures were used for genomic sequencing as described (*9*). Genomic fragment amplification was conducted by using reverse transcription PCR and 14 pairs of primers (*10*), which had minor modifications made on the basis of the genomic sequence of NADC30 available in GenBank. Comparative analyses of all coding regions and their deduced amino acid sequences of the virus were performed with representative PRRSV strains from China and the United States. Similar to the genome of NADC30, the genome of CHsx1401 (GenBank accession no. KP861625) was 15,020 nt, excluding its poly A tail.

Amino acid alignment of the nonstructural protein 2 (NSP2) highly variable region of CHsx1401 with those other strains showed that this virus had amino acid deletions that were identical to that in NADC30 (*8*) and MN184 isolated in the United States (*4*). These deletions were identified as a 111-aa deletion at position 323–433, a 1-aa deletion at position 481, and a 19-aa deletion at position 533–551 (Technical Appendix Figure) when compared with sequence of prototype strain VR-2332. 

Two recent virus isolates from China (HENAN-XINX and HENAN-HEB), whose sequences were submitted to GenBank in 2013, also had these deletions. Genome sequence of CHsx1401 had 95.7% nucleotide identity with NADC30, 93.0% identity with HENAN-XINX, and 93.2% identity with HENAN-HEB, but only 85.8% identity with VR-2332 and 83.8% identity with JXwn06, a highly pathogenic strain from China. Phylogenetic analysis of the whole genome of PRRSV was performed by using a distance-based neighbor-joining method with 1,000 bootstrap replicates in MEGA6 (http://www.megasoftware.net/). CHsx1401 was shown to be genetically more closely related to NADC30 and clustered into a specific branch (Figure).

**Figure:**
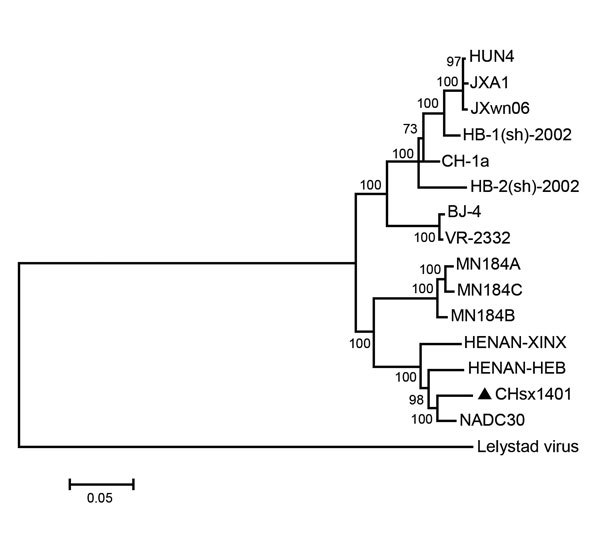
Phylogenetic analysis of whole genomes of porcine reproductive and respiratory syndrome virus (PRRSV) CHsx1401 (triangle) (GenBank accession no. KP861625); representative prototype strain VR-2332 (U87392); isolates BJ-4 (AF331831), CH-1a (AY032626), HB-1(sh)/2002 (AY150312), and HB-2(sh)/2002 (AY262352) from China; highly pathogenic strains JXA1 (EF112445), JXwn06 (EF641008), and HUN4 (EF635006); strains MN184A (DQ176019), MN184B (DQ176020), MN184C (EF488739), and NADC30 (JN654459) from the United States; and recent strains HENAN-HEB (KJ143621) and HENAN-XINX (KF611905) from China. Prototype Lelystad virus (M96262) was used as the outgroup. The phylogenetic tree was constructed by using the distance-based neighbor-joining method with 1,000 bootstrap replicates in MEGA6 (http://www.megasoftware.net/). Numbers along branches are bootstrap values. Scale bar indicates nucleotide substitutions per site.

Additional comparative analyses of viral protein amino acid sequences of CHsx1401 with those of NADC30, MN184A, MN184B, MN184C, JXwn06, and VR-2332 indicated that CHsx1401 had higher similarity with NADC30 (91.2%–99.1%) than with MN184 serial strains (78%–98.2%) and lower similarity with HP-PRRSV (JXwn06) from China and VR-2332 strains, except for NSP1α and NSP11 (Technical Appendix Table). These data also indicate that CHsx1401 is genetically similar to NADC30.

Recent widespread outbreaks of PPRS in China were associated with a novel NADC30-like strain of PPRSV. Whole genomic analysis showed that the strain differed from previously identified PRRSV strains in China, but had an overall genetic similarity and a unique deletion in the NSP2-coding region that was identical to that of NADC30, which originated in the United States. We propose that the NADC30 strain was introduced into China in recent years by importing of breeding pigs and has since undergone mutations, resulting in variant viruses.

**Technical Appendix.** Additional information on NADC30-like strain of porcine reproductive and respiratory syndrome virus, China.
