# Supplementary material for: NADC30-like Strain of Porcine Reproductive and Respiratory Syndrome Virus, China
Source: Emerg Infect Dis. 2015 Dec;21(12):2256–7. doi: 10.3201/eid2112.150360 (PMC4672414; doi:10.3201/eid2112.150360)
Supplement: Supplementary file 1 — Technical Appendix. Additional information on NADC30-like strain of porcine reproductive and respiratory syndrome virus, China. [file 15-0360-Techapp-s1.pdf]

Article DOI: http://dx.doi.org/10.3201/eid2112.150360

NADC30-like Strain of Porcine Reproductive and Respiratory Syndrome Virus, China

Technical Appendix

Additional information on NADC30-like strain of porcine reproductive and respiratory syndrome virus, China

Technical Appendix Table. Nucleotide and amino acid identities of viral proteins of porcine reproductive and respiratory syndrome virus CHsx1401 and other representative strains

	Isolate, country/year of isolation, % identity											
	NADC30, USA/2008		MN184A, USA/2001		MN184B, USA/2001		MN184C, USA/2001		JXwn06, China/2006		VR-2332, USA/1992	
Protein*	nt	aa	nt	aa	nt	aa	nt	aa	nt	aa	nt	aa
NSP1α	95.8	95.8	88.1	93.9	88.1	93.9	88.7	94.5	86.5	95.2	88.5	95.8
NSP1β	95.6	92.2	85.2	82.6	86.1	84.4	86.2	84.4	79.8	75.2	80.6	76.1
NSP2	94.0	91.2	82.9	78.2	83.7	79.5	83.4	78.8	74.4	66.9	77.2	70.4
NSp3	96.9	98.0	84.6	90.6	84.8	90.6	85.0	90.8	83.0	90.4	86.0	90.1
NSP4	96.1	97.5	82.7	91.7	83.2	93.6	82.7	91.7	84.5	93.6	85.1	93.1
NSP5	96.1	95.3	87.5	91.2	88.8	90.6	82.9	85.9	87.6	89.4	88.6	90.0
NSP6	89.6	93.8	83.3	93.8	83.3	93.8	79.2	93.8	83.3	87.5	83.3	93.8
NSP7	96.5	96.5	86.7	88.4	86.5	88.8	86.6	88.8	82.1	83.0	86.2	88.4
NSP8	98.6	97.8	93.5	95.7	92.8	93.5	93.5	95.7	89.9	93.5	91.3	93.5
NSP9	97.1	98.3	90.7	94.9	90.9	95.5	91.2	95.7	87.4	96.9	88.9	96.3
NSP10	96.3	99.1	92.5	98.0	92.3	98.0	92.4	98.2	85.1	95.2	85.8	95.5
NSP11	91.5	95.1	89.2	94.6	89.2	94.2	89.4	94.6	91.6	95.5	96.3	97.8
NSP12	95.5	95.5	85.1	90.9	85.1	90.9	85.1	90.9	88.1	94.8	87.0	92.9
ORF2a/GP2a†	95.6	95.3	85.9	86.8	85.9	86.4	85.9	86.8	86.9	87.5	87.9	90.3
ORF2b/E	95.0	91.9	91.9	91.9	91.9	91.9	91.4	91.9	91.0	87.8	90.5	86.5
ORF3/GP3	97.0	96.5	83.5	83.1	83.4	83.5	83.5	83.9	83.8	81.2	83.7	82.0
ORF4/GP4	96.6	97.8	89.4	88.8	89.4	88.3	89.6	88.8	85.3	88.8	86.8	87.2
ORF5/GP5	94.7	94.0	88.6	87.6	89.1	88.6	89.1	88.6	85.7	85.6	85.2	83.6
ORF6/M	97.3	97.7	88.8	94.9	88.8	94.9	89.1	94.9	88.8	93.1	89.9	92.6
ORF7/N	95.4	94.4	89.0	87.9	88.4	87.1	89.0	87.9	86.6	83.9	89.8	88.7

*NSP, nonstructural protein; ORF, open reading frame; GP, glycoprotein; E, envelope; M, membrane; N, nucleocapsid.
†Indicates genes of each open reading frame and their coded proteins, respectively.

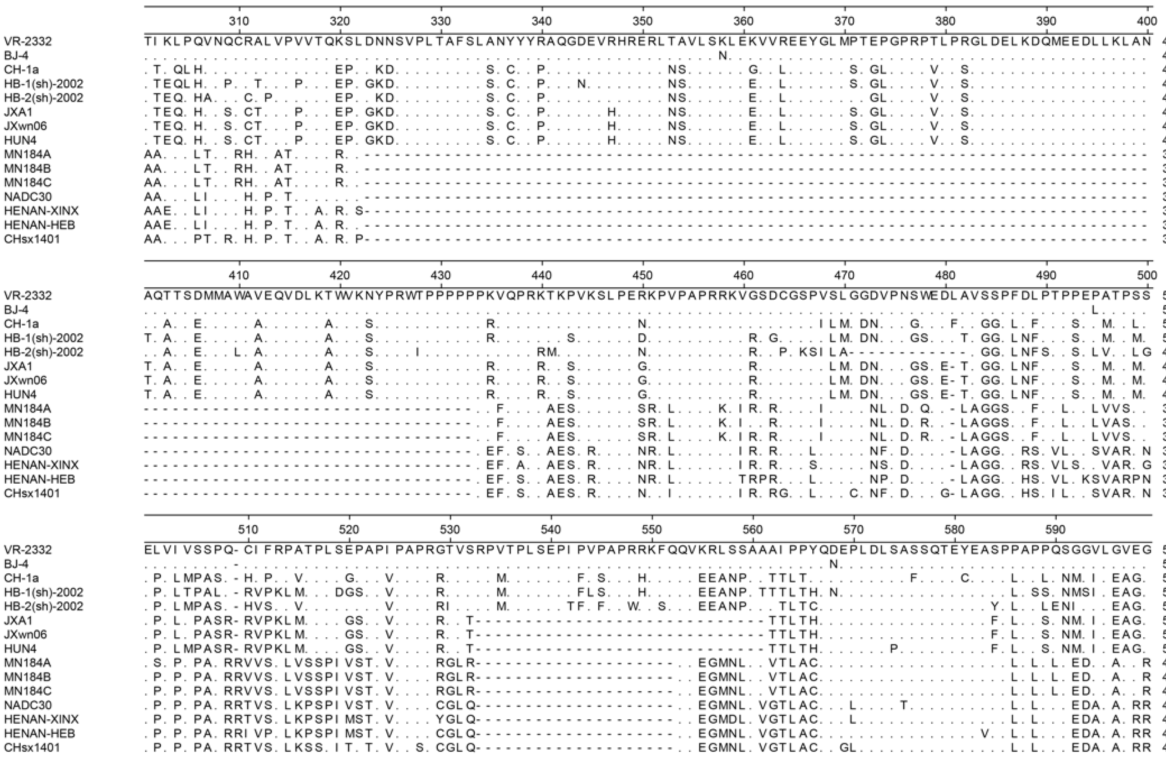

Technical Appendix Figure. Alignment of amino acid sequences of highly variable regions of nonstructural protein of porcine reproductive and respiratory syndrome viruses NADC30-like CHsx1401 (GenBank accession no. KP861625); representative prototype strain VR-2332 (U87392); isolates BJ-4 (AF331831), CH-1a (AY032626), HB-1(sh)/2002 (AY150312), and HB-2(sh)/2002 (AY262352) from China; highly pathogenic strains JXA1 (EF112445), JXwn06 (EF641008), and HUN4 (EF635006); strains MN184A (DQ176019), MN184B (DQ176020), MN184C (EF488739), and NADC30 (JN654459) from the United States; and recent strains HENAN-HEB (KJ143621) and HENAN-XINX (KF611905) from China. Dots indicate conserved residues, and hyphens dashes indicate deleted residues. Positions of deleted amino acids were determined on the basis of the genome of PRRVS strainVR-2332.
